# CCL2 promotes osteogenesis by facilitating macrophage migration during acute inflammation

**DOI:** 10.3389/fcell.2023.1213641

**Published:** 2023-06-30

**Authors:** Masakazu Toya, Ning Zhang, Masanori Tsubosaka, Junichi Kushioka, Qi Gao, Xueping Li, Simon Kwoon-Ho Chow, Stuart B. Goodman

**Affiliations:** ^1^ Department of Orthopaedic Surgery, Stanford University School of Medicine, Stanford, CA, United States; ^2^ Department of Orthopaedics and Traumatology, The Chinese University of Hong Kong, Shatin, Hong Kong SAR, China; ^3^ Department of Bioengineering, Stanford University, Stanford, CA, United States

**Keywords:** CCL2, MCP1, MSc, macrophage, osteogenesis, migration, inflammation

## Abstract

Novel minimally invasive strategies are needed to obtain robust bone healing in complex fractures and bone defects in the elderly population. Local cell therapy is one potential option for future treatment. Mesenchymal stromal cells (MSCs) are not only involved in osteogenesis but also help direct the recruitment of macrophages during bone regeneration via MSC-macrophage crosstalk. The C-C motif chemokine ligand 2 (CCL2) is an inflammatory chemokine that is associated with the migration of macrophages and MSCs during inflammation. This study investigated the use of CCL2 as a therapeutic target for local cell therapy. MSCs and macrophages were isolated from 10 to 12 week-old BALB/c male mice. Genetically modified CCL2 over-expressing MSCs were produced using murine CCL2-secreting pCDH-CMV-mCCL2-copGFP expressing lentivirus vector. Osteogenic differentiation assays were performed using MSCs with or without macrophages in co-culture. Cell migration assays were also performed. MSCs transfected with murine CCL2-secreting pCDH-CMV-mCCL2-copGFP expressing lentivirus vector showed higher levels of CCL2 secretion compared to unaltered MSCs (*p* < 0.05). Genetic manipulation did not affect cell proliferation. CCL2 did not affect the osteogenic ability of MSCs alone. However, acute (1 day) but not sustained (7 days) stimulation with CCL2 increased the alizarin red-positive area when MSCs were co-cultured with macrophages (*p* < 0.001). Both recombinant CCL2 (*p* < 0.05) and CCL2 released from MSCs (*p* < 0.05) facilitated macrophage migration. We demonstrated that acute CCL2 stimulation promoted subsequent osteogenesis in co-culture of MSCs and macrophages. Acute CCL2 stimulation potentially facilitates osteogenesis during the acute inflammatory phase of bone healing by directing local macrophage migration, fostering macrophage-MSC crosstalk, and subsequently, by activating or licensing of MSCs by macrophage pro-inflammatory cytokines. The combination of CCL2, MSCs, and macrophages could be a potential strategy for local cell therapy in compromised bone healing.

## Introduction

During the past few decades, many orthopaedic surgeries have been successfully developed leading to a numerous number of patients having been relieved of their disabilities by surgical interventions like total joint replacement. Meanwhile, older patients have substantially increased in recent years and so as the requirement for less invasive treatments. Because of the better establishment of stem cell research these days, local stem cell therapy is one of the most potent options for future treatment in bone or cartilage regeneration.

Mesenchymal stromal cells (MSCs), the precursor cells for bone and cartilage, are crucial for bone regeneration. MSCs differentiate into osteoblasts during intramembranous ossification and chondrocytes during endochondral ossification (Ito, 2011). MSCs are also involved in the recruitment of macrophages during fracture healing (Otsuru et al., 2008; Granero-Moltó et al., 2009). Crosstalk between MSCs and macrophages is critical for subsequent osteogenesis (Pajarinen et al., 2019). Different subsets of macrophages including the more commonly known M1 (pro-inflammatory) or M2 (anti-inflammatory) macrophages that secrete different sets of cytokines have previously been shown *in vitro* to regulate osteogenic differentiation in MSC (Lu et al., 2017) and bone formation capacity in osteoblasts (Loi et al., 2016). Macrophages were also shown *in vivo* to play important regulatory roles in bone regeneration in rodent fracture models where the depletion of macrophages (Schlundt et al., 2018) or the suppression of innate immune response by non-steroid anti-inflammatory drugs could lead to suboptimal healing of fracture (Chow et al., 2019). Furthermore, macrophages initiate the recruitment of MSCs and vascular progenitor cells from the periosteum, bone marrow, and circulation (Bastian et al., 2011; Wu and Zeng, 2013). Due to these multifaceted characteristics of the cell-cell interaction between MSCs and macrophages, they are a possible candidate for local cell therapy.

C-C motif chemokine ligand 2 (CCL2) is a member of the CC chemokine superfamily (Murphy, 1996). CCL2 is an inflammatory chemokine that regulates leukocyte recruitment during inflammatory responses (Murphy, 1996; Paavola et al., 1998; Hemmerich et al., 1999; Jarnagin et al., 1999). In the bone microenvironment, CCL2 is expressed by osteoblasts and promotes the subsequent recruitment and migration of macrophages and endothelial cells via binding to the C-C motif chemokine receptor 2 (CCR2) (Rahimi et al., 1995; Jiang and Graves, 1999). CCL2 could induce the recruitment of monocytes to the bone, and it is associated with an increase in osteoblast numbers (Posner et al., 1997). According to the accumulating evidence of CCL2 in the skeletal system, the impact of CCL2 on osteogenesis can not be overlooked.

We hypothesized that CCL2 is a prime mediator of cellular crosstalk between MSCs and macrophages and leads to improved osteogenesis. This study investigated the therapeutic potential of CCL2-mediated local cell treatment using genetically modified CCL2-releasing MSCs and recombinant CCL2 protein.

## Materials and methods

### Mice and cells

10–12 week-old BALB/c male mice (Jackson Laboratory, Bar Harbor, ME, United States) were used for primary cell culture. Mice were housed in a specific pathogen-free facility with a 12-h light, 12-h dark cycle and given free access to food and water. Bone marrow-derived mesenchymal stromal cells (MSCs) and bone marrow-derived macrophages were isolated from mice as previously described (Peister et al., 2004; Lin et al., 2015; Lin et al., 2019; Nathan et al., 2019; Zhang et al., 2021). Briefly, murine bone marrow was collected from femurs and tibias, then suspended and filtered through the 70 μm cell strainer, spun down, and resuspended in α-minimal essential medium (α-MEM, Thermo Fisher Scientific, Waltham, MA, United States) supplemented with 10% certified fetal bovine serum (FBS, Thermo Fisher Scientific) and 1% antibiotic and antimycotic solution (A/A, Thermo Fisher Scientific). The medium was replaced the next day to remove unattached cells. MSCs were characterized and identified by flow cytometry (Sca1+/CD105+/CD44+/CD45−/CD34−/CD11b−) at passage 4. MSCs passages 4-8 were used in the following experiments. To isolate macrophages, bone marrow was also collected with the same procedures with MSCs, and resuspended in RPMI 1640 medium (Thermo Fisher Scientific) supplemented with 10% FBS and 1% A/A. After centrifugation, cells were resuspended in 1 mL of RBC lysis buffer (MilliporeSigma, Burlington, MA, United States) and centrifuged. Then, cells were resuspended in the augmented basal macrophage medium; RPMI 1640, 30% L929 leucocyte-conditioned medium, 10% FBS, 1% A/A, and 10 ng/mL macrophage colony-stimulating factor (M-CSF, R&D systems, Minneapolis, MN, United States), and cultured for 5 days to obtain naïve primitive macrophages (M0). The animal experimental protocol was reviewed and approved by Stanford’s Administrative Panel on Laboratory Animal Care (protocol number: APLAC-9964). Institutional Guidelines for the Care and Use of Laboratory Animals were followed in all aspects of this project. All studies were carried out in compliance with the ARRIVE guidelines.

### Construction of murine CCL2 plasmid

The constitutive murine C-C motif chemokine ligand 2 (CCL2) expression lentivirus driven by cytomegalovirus (CMV) promoter was released from the CCL2 expression pCMV-mCCL2-His plasmid (Sino Biological Inc., Beijing, China) by digestion with Spel/NotI restriction enzyme and ligated into the pCDH-CMV-copGFP lentiviral expression vector (CD511B-1; System BioSciences, Palo Alto, CA, United States) to generate the pCDH-CMV-mCCL2-copGFP vector.

### Generation of genetically modified MSCs

**FIGURE 1:**
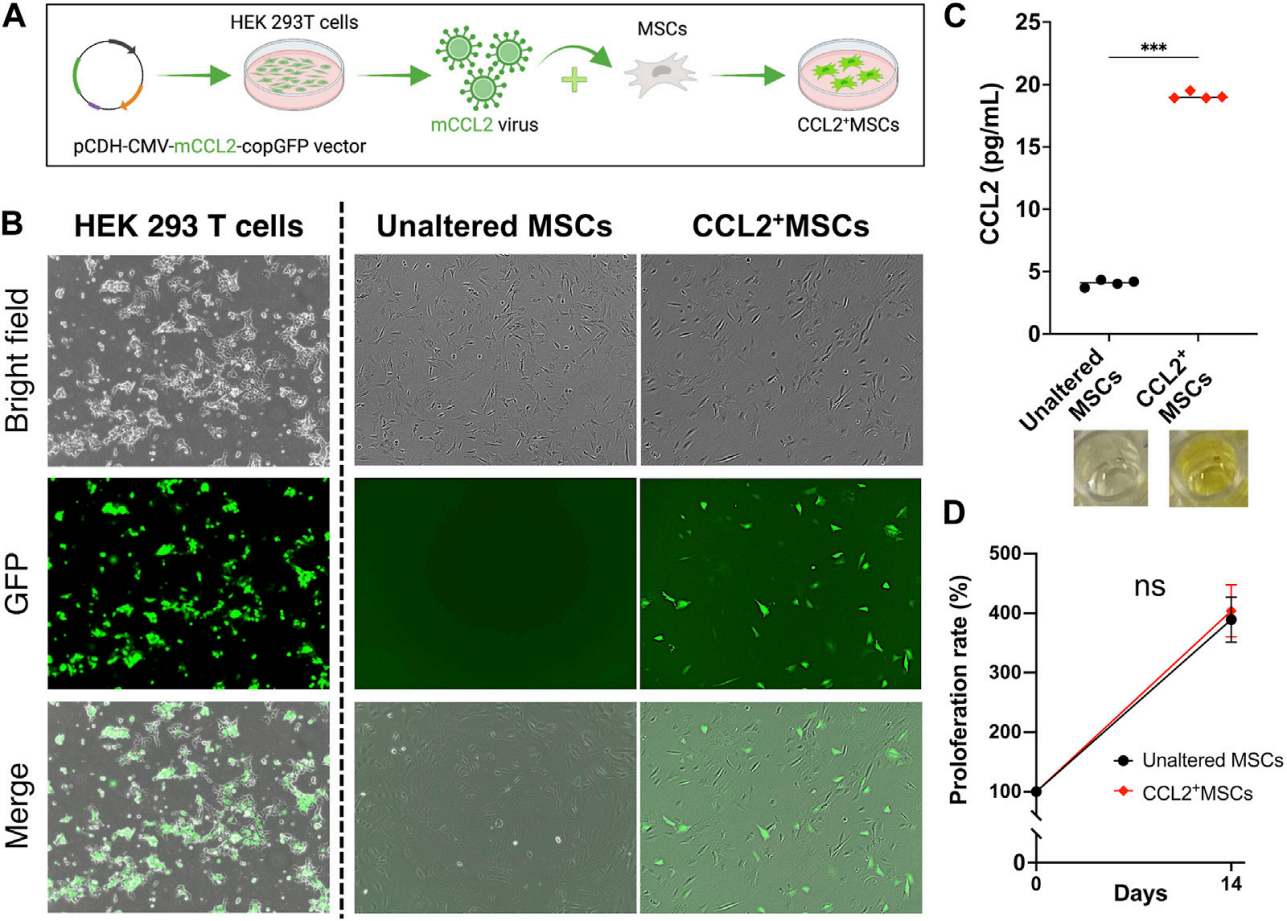
The establishment of genetically modified CCL2-releasing MSCs. **(A)** Human embryonic kidney 293T cells were used to co-transfect the murine CCL2 secreting pCDH-CMV-mCCL2-copGFP expressing lentivirus vector. The virus was diluted in MSCs’ culture medium and infected to MSCs. **(B)** The virus-infected cells were confirmed with GFP positive by fluorescence microscope. **(C)** Quantitative analysis of Enzyme-linked immunosorbent assay (ELISA). The supernatant was collected and diluted (1:1,000). (N = 4, each group, the Welch test, *** = *p* < 0.001) **(D)** The comparison of cell proliferation rate; unaltered control MSCs (Unaltered MSCs) (black), genetically modified CCL2-releasing MSCs (CCL2^+^MSCs) (red).

The lentiviral vector preparation was performed as previously described (Pajarinen et al., 2015; Zhang et al., 2021). Human embryonic kidney 293T cells (ATCC, Manassas, VA, United States) were used to transfect the murine CCL2 secreting pCDH-CMV-mCCL2-copGFP expressing lentivirus vector together with psPAX2 packaging vector and pMD2G VSV-G envelope vector using the calcium phosphate transfection kit (Clontech, Mountain View, CA, United States) with 25 μM chloroquine. The lentivirus vector pCDH-CMV-copGFP (CD511B-1; System Biosciences, Palo Alto, CA) was used to generate the empty control virus. The virus was diluted in MSCs’ culture medium supplemented with 6 μg/mL of polybrene (Sigma Aldrich, St. Louis, MO, United States) and infected to murine MSCs (Figure 1A) after virus titration at the multiplicity of infection (MOI) = 100. The virus-infected cells were confirmed with GFP positive by fluorescence microscope (BZ-X810, KEYENCE, Osaka, Japan) 3 days after infection (Figure 1B).

### Enzyme-linked immunosorbent assay (ELISA)

To confirm the success of genetic modification, ELISA was performed. Briefly, collected cells were seeded into the 24-well plate and incubated for 24 h, then the supernatant was collected from each well and diluted (1:1,000). The secretion level of CCL2 was evaluated using DuoSet (R&D systems) according to the manufacturer’s protocol. Optical densities were measured using SpectraMax iD3 (Molecular Devices, San Jose, CA, United States).

### Cell viability and proliferation assay

Cell viability and proliferation were evaluated using alamarBlue Cell Viability Reagent (Thermo Fisher Scientific) as previously described (Al-Nasiry et al., 2007; Rampersad, 2012; Longhin et al., 2022). Assays were conducted according to the manufacturer’s protocol. Briefly, cells were seeded and cultured with the 96-well plate, then 10 μL of alamarBlue reagent was added to each well and incubated for 1 h at 37°C. The fluorescence was measured using SpectraMax iD3 (Molecular Devices).

### Cell culture

**FIGURE 2:**
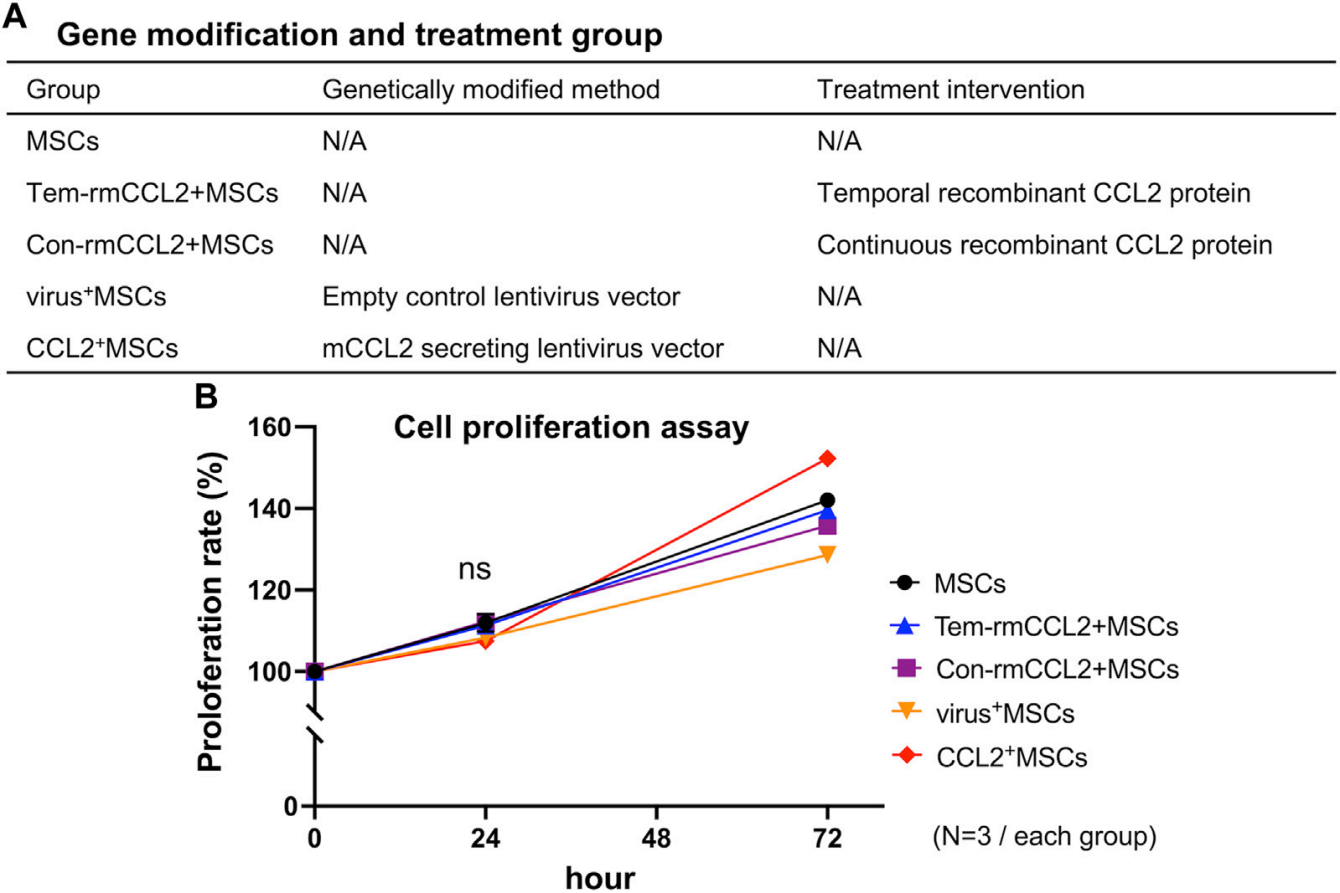
Treatment groups and cell proliferation. **(A)** The explanation of each group in terms of genetic modification and treatment intervention. **(B)** The comparison of cell proliferation rates among the 5 groups in Figure 2A. (N = 3, each group, 0.16 < S.D. < 2.12).

MSCs were seeded and cultured with or without recombinant murine CCL2 (10 ng/mL, rmCCL2, R&D systems) (Figure 2A); (1) MSCs, without any intervention; (2) Tem-rmCCL2+MSCs, unaltered control MSCs incubated with temporal rmCCL2 stimulation for the initial 1 day of assay; (3) Con-rmCCL2+MSCs, unaltered control MSCs incubated with continuous rmCCL2 stimulation for the whole period of assay; (4) virus^+^MSCs, MSCs infected with empty lentivirus vector; (5) CCL2^+^MSCs, MSCs infected with murine CCL2 secreting lentivirus vector. These groups of MSCs were used for the following experiments. In addition, direct co-culture of MSCs with naïve primitive macrophages (1:1 ratio mix of each type of cells and basal medium) was also performed to investigate the effect of CCL2 on the cell-cell interaction between MSCs and macrophages.

### Osteogenic differentiation assay

Cells were seeded in the 24-well plate and cultured with the osteogenic medium; α-MEM (Thermo Fisher Scientific) supplemented with 10% certified fetal bovine serum (FBS, Thermo Fisher Scientific), 1% antibiotic and antimycotic solution (A/A, Thermo Fisher Scientific), 10 mM β-glycerol phosphate (Sigma-Aldrich), 50 μM 1 ascorbic acid (Sigma-Aldrich), and 100 nM dexamethasone (Sigma Aldrich). Alkaline phosphatase (ALP) staining was performed on day 7 using 1-Step^TM^ NBT/BCIP Substrate Solution (Thermo Fisher Scientific), and Alizarin Red staining (pH 4.1, Sigma Aldrich) for calcified bone matrix (Gregory et al., 2004) was performed on day 21. The deposition of Alizarin Red under mineralization conditions suggests that bone matrix has been deposited. Whole-well images were captured using BZ-X810 (KEYENCE), and ALP and Alizarin Red positive areas were measured using QuPath (Bankhead et al., 2017).

### Migration assay

The cell migration ability of macrophages was evaluated by scratch assay. M0 macrophages were cultured with recombinant CCL2 protein (10 ng/mL) or co-cultured with MSCs as described above. Briefly, cells were seeded in the 24-well plate and incubated overnight, then the bottom of each well was scratched once using 200 μL micropipettes (Kauanova et al., 2021). After the scratch, the progress of cell migration was captured at different time points using BZ-X810 (KEYENCE). The cell’s empty area and the distance between its area were measured using QuPath. The distance between the area was measured at 3 different places; the top, center, and bottom of each captured image, and averaged as a distance between edge to edge. The empty area and distances were measured using QuPath.

### Statistical analysis

All experiments were repeated at least three times. The Welch test was used for performing comparisons between two groups. The Kruskal-Wallis test with Dunn’s multiple comparisons was used for multiple non-parametric comparisons of greater than two groups. Data were expressed as median with standard deviation. All analyses were performed using Prism 9 (GraphPad Software, San Diego, CA, United States). *p* values less than 0.05 were considered significant.

## Results

### The proliferation of MSCs was not affected by CCL2 transfection

MSCs transfected murine CCL2 secreting pCDH-CMV-mCCL2-copGFP expressing lentivirus vector showed significantly greater secretion of CCL2 compared to unaltered control MSCs (*p* < 0.001) (Figure 1C). There was no significant difference between genetically modified CCL2^+^MSCs and unaltered control MSCs with respect to cell proliferation (Figure 1D). Also, recombinant murine CCL2 and CCL2 released from genetically modified MSCs did not affect subsequent cell proliferation (Figure 2B). These results were consistent with our previous report indicating that viral transfected MSCs showed no differences in proliferative capacity compared to the unaltered control MSCs (Zhang et al., 2021).

### The osteogenic ability of MSCs was not affected by CCL2 transfection

**FIGURE 3:**
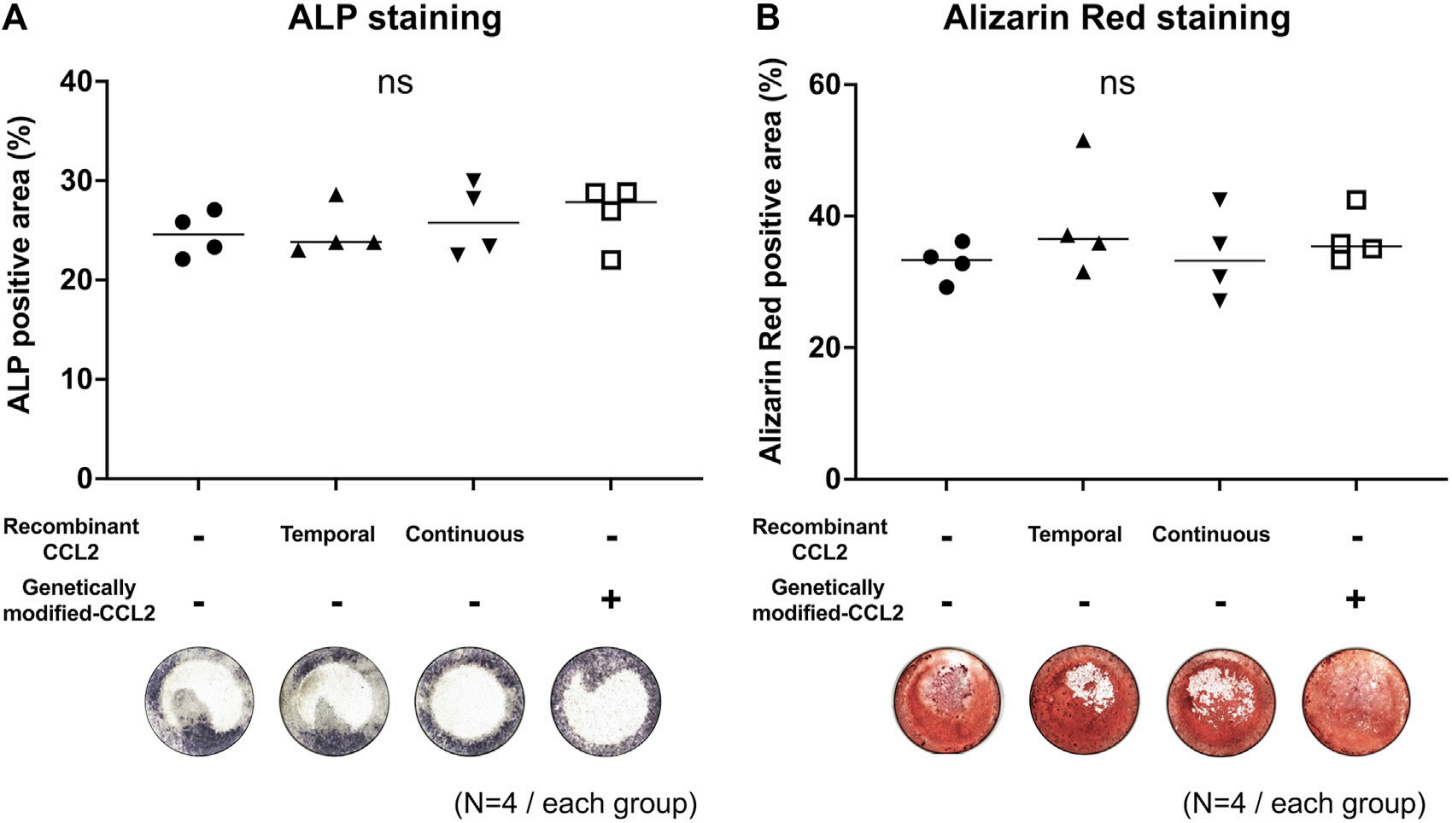
Mono-culture of MSCs; osteogenic differentiation assay. Osteodifferentiation assays were performed using MSCs. Cells were seeded in the 24-well plate and cultured. Unaltered MSCs were cultured with or without recombinant CCL2 protein for 1 day (Temporal) or the whole culture period (Continuous). Alkaline phosphatase (ALP) staining was performed on day 7, and Alizarin Red staining on day 21. Quantitative analysis of **(A)** ALP and **(B)** Alizarin Red positive area proportion (%/well) and representative whole-well images of each group were shown. (N = 4, each group, the Kruskal-Wallis test).

Different MSC groups were cultured in osteogenic differentiation medium. Since we showed that lentivirus itself did not affect the MSCs proliferation and osteogenic proliferation, the virus^+^MSCs group was excluded from the following experiments (Supplementary Material). As a result, all groups had no significant differences in osteogenic ability (Figure 3). Both temporal and continuous stimulation with recombinant CCL2 did not affect subsequent osteogenesis. Also, CCL2 released from genetically modified MSCs did not affect the osteogenic differentiation ability of MSCs.

### Temporal, but not continuous stimulation of CCL2 enhanced osteogenesis in MSC-macrophage co-culture

**FIGURE 4:**
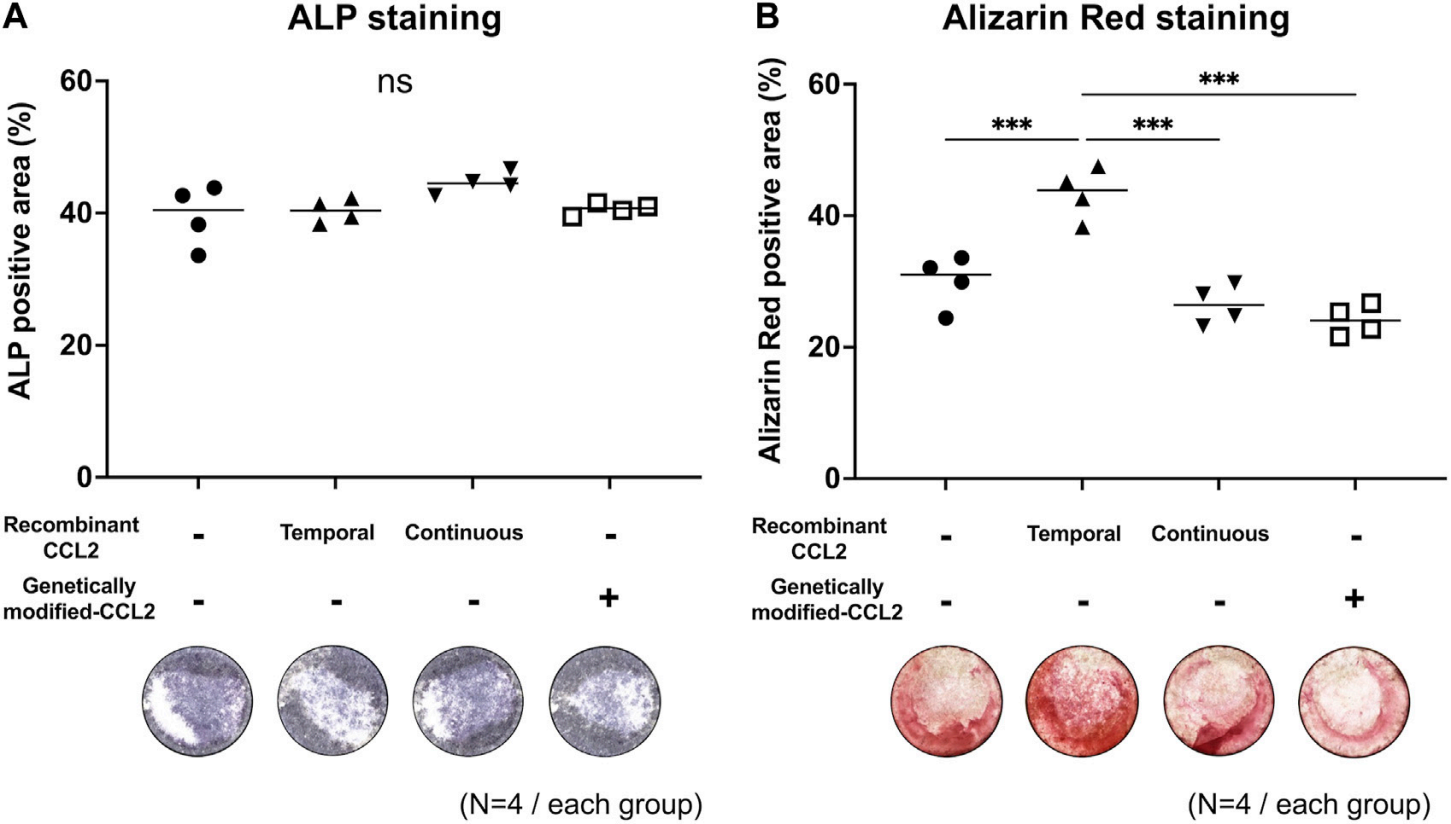
Co-culture of MSCs with macrophages; osteogenic differentiation assay. Osteodifferentiation assays were performed by direct co-culture of MSCs with naïve primitive macrophages (1:1 ratio mix of each type of cells and basal medium). Cells were seeded in the 24-well plate and co-cultured. Unaltered MSCs and macrophages were cultured with or without recombinant CCL2 protein for 1 day (Temporal) or the whole culture period (Continuous). Alkaline phosphatase (ALP) staining was performed on day 7, and Alizarin Red staining on day 21. Quantitative analysis of **(A)** ALP and **(B)** Alizarin Red positive area proportion (%/well) and representative whole-well images of each group were shown. (N = 4, each group. The Kruskal-Wallis test, *** = *p* < 0.001).

To examine the effect of CCL2 on the interaction between MSCs and macrophages, MSCs were co-cultured with macrophages, and osteogenic differentiation was induced by using a combined medium. ALP-positive area was comparable among all groups (Figure 4A). The group treated with recombinant CCL2 for the initial 1 day showed a greater Alizarin Red-positive area than the other groups (*p* < 0.001), including continuous CCL2 stimulation groups (Figure 4B).

### Both recombinant CCL2 and CCL2 released from MSCs facilitate the migration of macrophages

**FIGURE 5:**
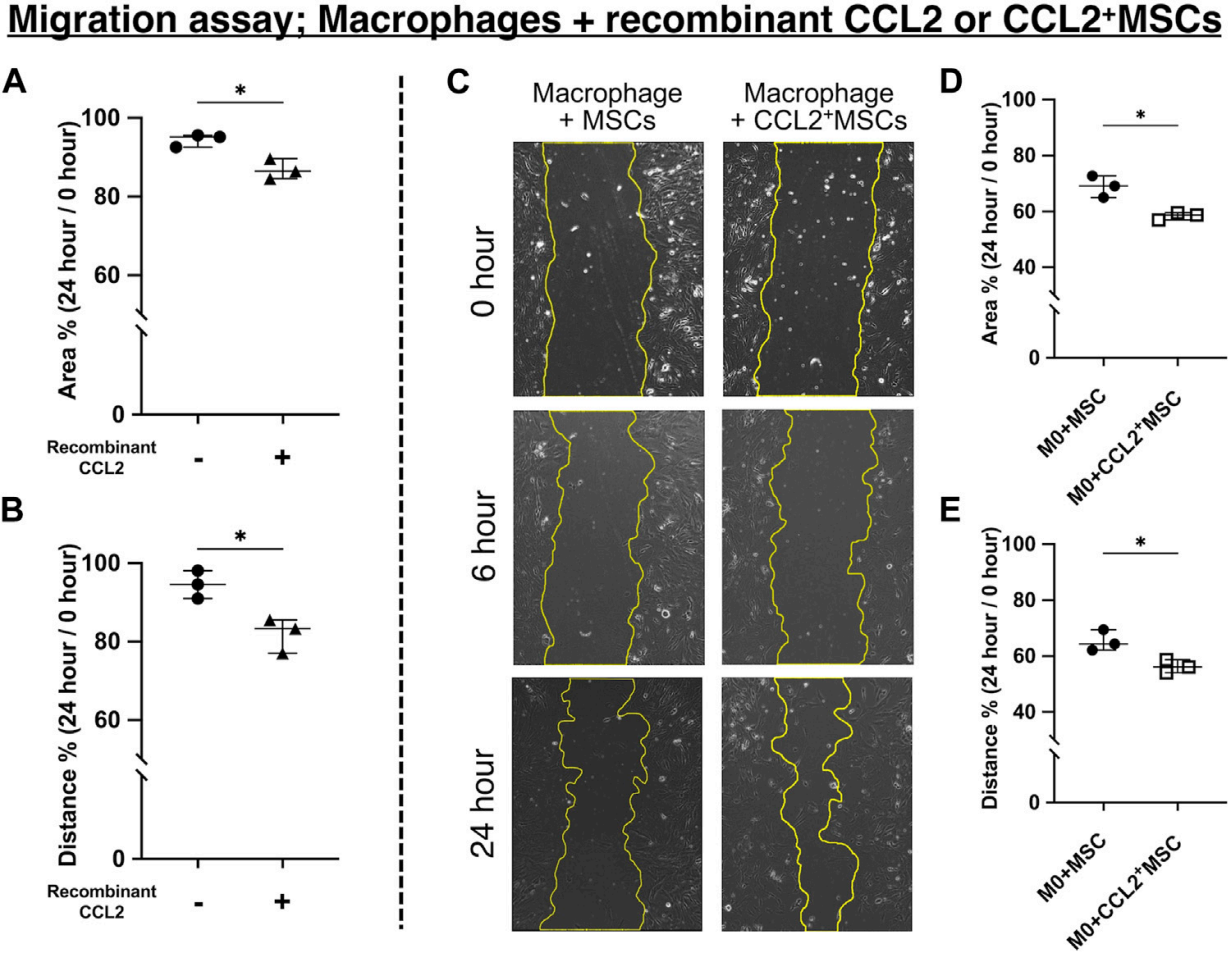
Cell migration assay. **(A, B)** Quantitative analysis of cell migration assay using macrophages and recombinant CCL2 protein. Measured **(A)** area and **(B)** distance 24 h after scratching the bottom of the well. The ratio was calculated compared to the area just after scratching. (N = 3, each group, the Welch test, * = *p* < 0.05) **(C–E)** Quantitative analysis of cell migration assay using macrophages co-cultured with CCL2^+^MSCs or unaltered MSCs. **(C)** Representative images of the scratched area at different time points in each group. Measured **(D)** area and **(E)** distance 24 h after scratching the bottom of the well. The ratio was calculated compared to the area just after scratching. (N = 3, each group, the Welch test, * = *p* < 0.05).

To investigate the potential role of CCL2 in osteogenesis, we performed the cell migration assay using the scratch test. After 24 h of incubation, the scratched area was decreased in the macrophages group incubated with recombinant CCL2 compared to non-treated macrophages (*p* < 0.05) (Figure 5A). The average distance from edge to edge of the scratched area was also shorter in the CCL2-treated macrophages group (*p* < 0.05) (Figure 5B). These results indicate that recombinant CCL2 promoted macrophage migration. Macrophages were also co-cultured with MSCs or CCL2-releasing MSCs. In both groups, the cells gradually migrated into the scratched area (Figure 5C). After 24 h, genetically modified CCL2-releasing MSCs exhibited greater cell migration ability than non-modified MSCs (*p* < 0.05) (Figures 5D, E).

## Discussion

Fragility fractures, nonunions, and bone defects in the elderly are unsolved clinical problems. In this regard, local autologous cell therapy is one potential strategy to obtain more robust and expedited union of difficult fractures and bone defects in elderly patients and others with compromised healing. MSCs are the precursors for osteoblasts, chondrocytes, and other cells that are essential for bone healing. Crosstalk between mesenchymal lineage cells and immune cells such as macrophages is also important for subsequent osteogenesis (Pajarinen et al., 2019). Macrophages initiate the recruitment of MSCs and vascular progenitor cells from the periosteum, bone marrow, and circulation (Kumagai et al., 2008; Colnot, 2009; Bastian et al., 2011; Wu and Zeng, 2013). Due to these multifaceted characteristics, MSCs are a possible candidate for local cell therapy.

CCL2, also called monocyte chemoattractant protein-1 (MCP-1) is an inflammatory chemokine that regulates leukocyte recruitment during inflammation. CCL2 binds to the C-C motif chemokine receptor 2 (CCR2) to induce chemotactic activity and increase calcium influx (Paavola et al., 1998; Hemmerich et al., 1999; Jarnagin et al., 1999). Because the interaction of CCL2 and CCR2 activates important inflammatory signal cascades, including PI3K/Akt/ERK/NF-κB, PI3K/MAPKs, and JAK/STAT-1/STAT-3 (Zhu et al., 2021), CCL2 and CCR2 have been proposed as potential therapeutic targets for a range of human diseases. For example, recombinant CCL2 was found to induce aortic valve interstitial cell (AVIC) calcification, with calcium deposition accompanied by osteoblastic transformation and increased phosphorylated-Akt expression; the knockdown of CCR2 attenuated AVICs’ osteoblastic transformation and calcification (Zhu et al., 2019). In orthopaedics, chronic inflammation is also associated with numerous orthopaedic conditions including rheumatoid arthritis (RA) (Ji et al., 2021) and osteoarthritis (OA) (Robinson et al., 2016). Modulation of CCL2 signaling by CCR-2B antagonists and CCL2-blocking antibodies in an experimental model of RA showed beneficial effects on inflammation and joint destruction (Dawson et al., 2003). However, subsequent clinical trials were unable to support the findings from animal studies; the blockade of CCR2 was ineffective in producing clinical improvement in RA patients (Vergunst et al., 2008). In addition, a randomized clinical trial of human anti-CCL2 monoclonal antibody (ABN912) treatment also concluded that ABN912 did not result in clinical or immunohistologic improvement (Haringman et al., 2006). Targeting of CCL2 and CCR2 might be a potential treatment of osteoarthritis (OA); *in vivo* findings from a murine OA model indicated that selective targeting of CCL2/CCR2, rather than CCL5/CCR5, was a viable therapeutic strategy (Raghu et al., 2017). However, pharmacologic inhibition of CCL2 by RS504393 also blocked type II collagen and aggrecan cleavage induced by TGF-α in a rodent OA model (Appleton et al., 2015). Therefore, the clinical benefit of CCL2 blockade in mitigating inflammation in musculoskeletal diseases is still controversial.

Accumulating evidence has indicated that CCL2 is an important regulator of the skeletal system (Siddiqui and Partridge, 2017; Rana et al., 2018). In human studies, CCL2 and CCR2 gene variants were identified as potential risk factors for osteoporosis and osteopenia (Eraltan et al., 2012). Osseous inflammation in the murine mandible found that CCL2 expressed by osteoblasts is an important effector of the monocyte/macrophage recruitment (Rahimi et al., 1995). CCL2 expressed by osteoblasts facilitates subsequent recruitment and migration of mononuclear cells and endothelial cells via binding to CCR2. CCL2 expression in osteoblasts and mononuclear cells is induced by lipopolysaccharide (LPS) under inflammatory conditions (Jiang and Graves, 1999). CCL2 induces the recruitment of monocytes and is associated with an increase in osteoblast numbers (Posner et al., 1997), and bone formation/resorption in a site-specific manner (Volejnikova et al., 1997). The expression of CCL2 in osteoblasts appears to mediate the paracrine recruitment of monocytes during bone remodeling, and CCL2 expression by osteoblasts is upregulated in response to inflammatory stimuli (Graves et al., 1999b; a).

CCL2 also affects immune cells including macrophages. Transgenic expression of CCL2 in adipose tissue increases macrophage infiltration, inflammation, and insulin resistance, whereas knockdown of CCL2 or CCR2 impairs the migration of macrophages into adipose tissue (Odegaard et al., 2007). CCL2 deficiency also increases anti-inflammatory macrophages (M2) polarization and decreases pro-inflammatory macrophage (M1) polarization, leading to increased browning of adipocytes (Rajasekaran et al., 2019). CCL2 is a major contributor to synovial tissue degradation in RA via the regulation of lymphocyte and monocyte/macrophage migration, the stimulation of synovial cells, and angiogenesis (Nanki, 2016). In an adjuvant-induced arthritis model of RA, CCR2, and the expression of other chemokines were increased, accompanied by increased activation of JAK/STAT1/STAT3 pathways, as well as macrophage and endothelial cell infiltration (Shahrara et al., 2003). These results suggest that a sustained level of CCL2 in adipose metabolism, or RA is both undesirable because it will maintain the inflammatory stage and hinder the resolution (lower M2).

**FIGURE 6:**
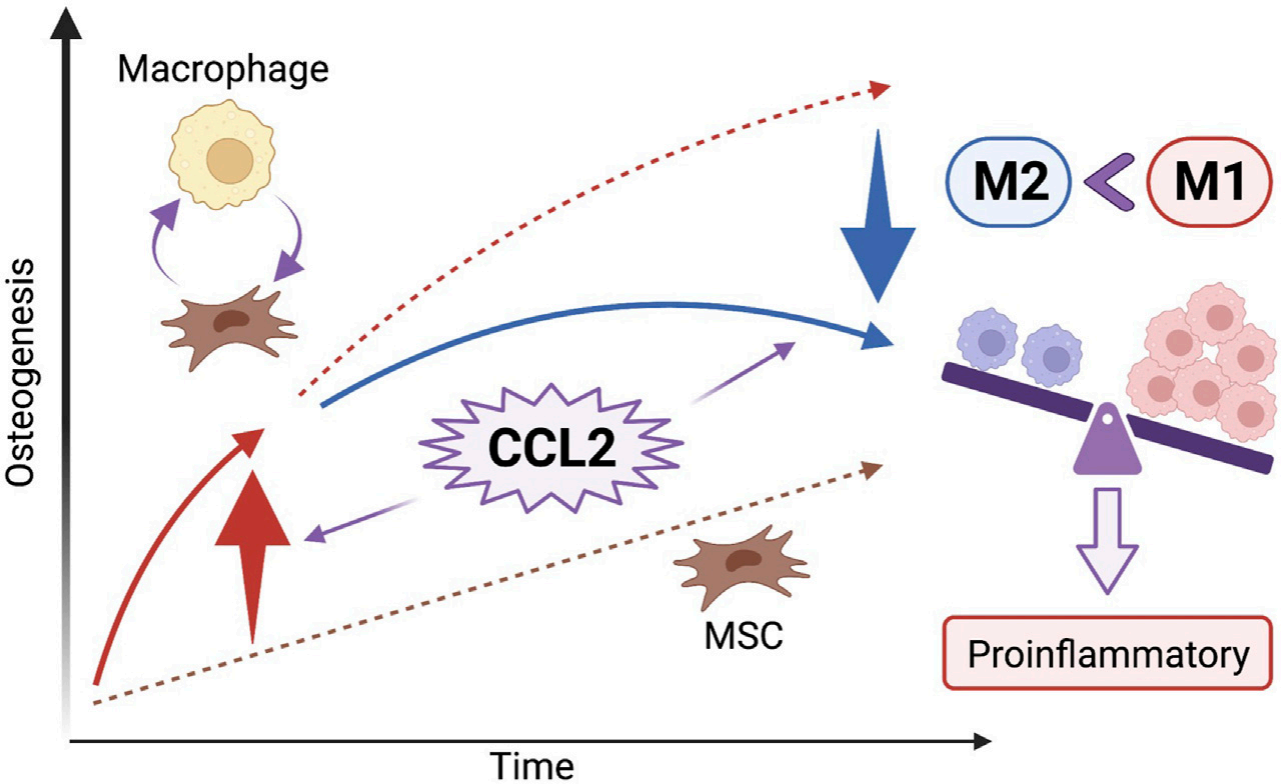
The effect of CCL2 stimulation on osteogenesis and the cell-cell interaction between MSCs and macrophages. CCL2 stimulation at the initial stage of inflammation facilitates the chemotaxis of macrophages and the cell-cell interaction between MSCs and macrophages resulting in greater osteogenesis. However, continuous CCL2 stimulation might foster prolonged pro-inflammatory conditions; an increase of pro-inflammatory M1 macrophages ratio resulting in bone loss rather than osteogenesis because of chronic inflammation.

In the present study, we demonstrated that acute stimulation with CCL2 for 1 day accelerated the migration of macrophages and facilitated subsequent osteogenesis. While both recombinant CCL2 and CCL2 released from genetically modified MSCs did not affect cell proliferation and osteogenesis of MSCs alone, when MSCs were co-cultured with macrophages, osteogenesis was promoted only with acute CCL2 stimulation. In the cell migration assay, both recombinant CCL2 and CCL2 released from MSCs facilitated the migration of macrophages. Our results revealed that acute CCL2 for 1 day, but not continuous stimulation for 7 days, induced macrophage migration and greater osteogenesis. One possibility is that continuous stimulation with CCL2 would increase M1 pro-inflammatory and decrease M2 pro-reconstructive macrophage polarization (Rajasekaran et al., 2019). Our results indicate that CCL2 plays a crucial role in the chemotaxis of macrophages at the initial stage of inflammation, but continuous CCL2 stimulation might foster prolonged pro-inflammatory conditions, and decreased osteogenesis (Figure 6).

The current study has limitations. First, this study describes *in vitro* experiments; further, *in vivo* studies are desirable to translate CCL2-releasing MSCs into the clinical situation. Second, the genetically modified CCL2-releasing MSCs in this study continuously released CCL2; additional gene modifications which can control the release of CCL2 may be beneficial for osteogenesis. Moreover, local optimal temporal delivery of CCL2 by other technologies, such as by scaffolds may be another option. Third, further investigations to include osteoclastogenesis would reveal the prospect of CCL2 for bone formation and degradation, processes critical for bone homeostasis.

In conclusion, we elucidated that acute (1 day) but not continuous CCL2 exposure facilitated subsequent osteogenesis in co-culture with MSCs and macrophages using genetically modified CCL2-releasing MSCs, recombinant CCL2, and macrophages. Both recombinant CCL2 and CCL2 secretion from MSCs accelerated macrophage migration during the initial stage of the inflammation. The addition of CCL2 acutely generates increased crosstalk between MSCs and macrophages and improves subsequent osteogenesis.

## Data Availability

The original contributions presented in the study are included in the article/Supplementary Material, further inquiries can be directed to the corresponding author.
