# Supplementary material for: CCL2 promotes osteogenesis by facilitating macrophage migration during acute inflammation
Source: Front Cell Dev Biol. 2023 Jun 30;11:1213641. doi: 10.3389/fcell.2023.1213641 (PMC10348816; doi:10.3389/fcell.2023.1213641)
Supplement: Supplementary file 1 [file DataSheet1.PDF]

Supplementary information

Title: CCL2 promotes osteogenesis by facilitating macrophages migration during acute inflammation.

Supplemental Figure S1. Osteogenic differentiation assay for Alizarin Red staining.

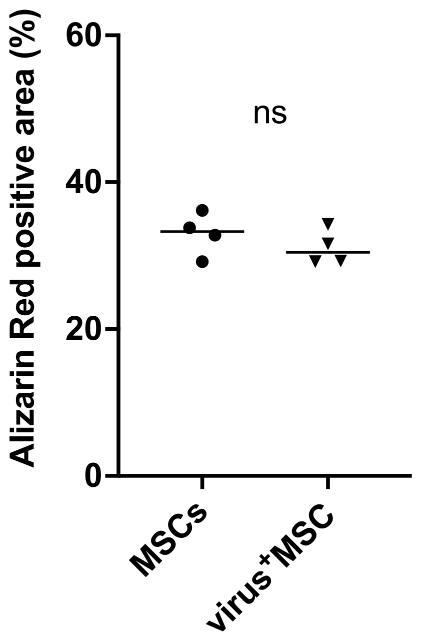

Osteodifferentiation assays were performed using MSCs. Cells were seeded in the 24-well plate and cultured. Unaltered MSCs were cultured with or without recombinant CCL2 protein for 1 day (Temporal) or the whole culture period (Continuous). Alizarin Red staining was performed on day 21. Quantitative analysis of Alizarin Red positive area proportion (% /well) was shown. (N=4, each group, the Mann-Whitney test)
